# Amorphous silica nanoparticles accelerated atherosclerotic lesion progression in ApoE^−/−^ mice through endoplasmic reticulum stress-mediated CD36 up-regulation in macrophage

**DOI:** 10.1186/s12989-020-00380-0

**Published:** 2020-10-02

**Authors:** Ru Ma, Yi Qi, Xinying Zhao, Xueyan Li, Xuejing Sun, Piye Niu, Yanbo Li, Caixia Guo, Rui Chen, Zhiwei Sun

**Affiliations:** 1 Department of Occupational Health and Environmental Health, School of Public Health, Capital Medical University, Beijing, 100069 China; 2 Beijing Key Laboratory of Environmental Toxicology, Capital Medical University, Beijing, 100069 China; 3 Department of Toxicology and Sanitary Chemistry, School of Public Health, Capital Medical University, Beijing, 100069 China

**Keywords:** Silica nanoparticles, Atherosclerosis, Foam cell, Endoplasmic reticulum stress, CD36

## Abstract

**Background:**

The biosafety concern of silica nanoparticles (SiNPs) is rapidly expanding alongside with its mass production and extensive applications. The cardiovascular effects of SiNPs exposure have been gradually confirmed, however, the interaction between SiNPs exposure and atherosclerosis, and the underlying mechanisms still remain unknown. Thereby, this study aimed to explore the effects of SiNPs on the progression of atherosclerosis, and to investigate related mechanisms.

**Results:**

We firstly investigated the in vivo effects of SiNPs exposure on atherosclerosis via intratracheal instillation of ApoE^−/−^ mice fed a Western diet. Ultrasound microscopy showed a significant increase of pulse wave velocity (PWV) compared to the control group, and the histopathological investigation reflected a greater plaque burden in the aortic root of SiNPs-exposed ApoE^−/−^ mice. Compared to the control group, the serum levels of total triglycerides (TG) and low-density lipoprotein cholesterol (LDL-C) were elevated after SiNPs exposure. Moreover, intensified macrophage infiltration and endoplasmic reticulum (ER) stress was occurred in plaques after SiNPs exposure, as evidenced by the upregulated CD68 and CHOP expressions. Further in vitro, SiNPs was confirmed to activate ER stress and induce lipid accumulation in mouse macrophage, RAW264.7. Mechanistic analyses showed that 4-PBA (a classic ER stress inhibitor) pretreatment greatly alleviated SiNPs-induced macrophage lipid accumulation, and reversed the elevated CD36 expression induced by SiNPs.

**Conclusions:**

Our results firstly revealed the acceleratory effect of SiNPs on the progression of atherosclerosis in ApoE^−/−^ mice, which was related to lipid accumulation caused by ER stress-mediated upregulation of CD36 expression in macrophage.

**Graphical abstract:**

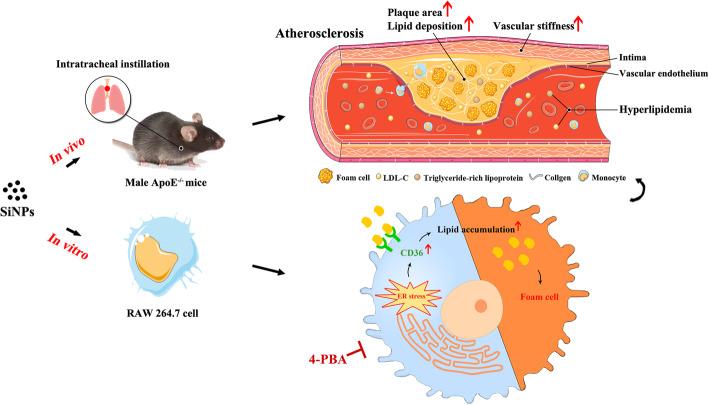

## Introduction

The rapid development and enormous progress in nanotechnology bring the toxicological concerns to nanomaterials (NMs), which might pose potential threats to human health and the environment. Silica nanoparticles (SiNPs) rank in the top two global productions in NMs, with an annual output of nearly 1.5 million tons [1]. It has a wide range of applications for industrial products and consumers, such as food additive, surfactants, catalysts, sensors, ceramics, paints, and also for medical and biomedical fields, e.g., drug delivery, disease diagnosis and treatment [2, 3]. Such mass production and widespread application of SiNPs would inevitably increase human exposure via occupational, environmental or even iatrogenic ways. Besides, SiNPs could enter into the natural environment through dust, construction, fuel combustion, etc., due to silicon is one of the most abundant minerals on Earth [4, 5]. Nevertheless, there is still a lack of biosafety data related to SiNPs, and discrepancy exists in acquired toxicological evidence, probably owing to different exposure scenarios (e.g., in vivo vs in vitro, acute vs chronic) [6, 7].

Despite the most important route of exposure to SiNPs being inhalation, especially in occupational settings, cardiovascular system is a principal site of extra-pulmonary toxic effects of SiNPs [8]. It has been well-documented that the inhaled nanoparticles (NPs) can translocate in the body, and NPs may act on the cardiovascular system in this process [9, 10]. We previously detected an increased silicon content in both serum and heart of SiNPs-treated rats via intratracheal instillation [8]. Intriguing, SiNPs were detected in the serum of systemic sclerosis patients with occupational exposure to silica dust [11]. Research also pointed out that inhaled NPs could translocate into the atherosclerotic plaques of mouse aortic arch and human carotid artery [12]. In despite of cardiovascular system as an important toxic site of NPs exposure, the current knowledge regarding the bio-toxic impact of engineered NPs on the cardiovascular system has not been fully clarified, and the potential toxic mechanisms are still being questioned [13]. To date, some occupational evidence has documented the adverse cardiovascular effects caused by engineered NPs exposure. For instance, Zhao et al. reported a correlation between cardiovascular disease markers (VCAM-1, ICAM-1, LDL, and TC) and the occupational exposure to titanium dioxide (TiO_2_) NPs [14]. Similarly, the association between multi-walled carbon nanotubes (MWCNTs) and ICAM-1 was also revealed, indicating endothelial activation in workers with MWCNTs exposure [15]. It was found that the prevalence of angina was significantly higher in NMs handling workers than in the controls [16], and cardiovascular markers (VCAM-1, ICAM-1 and low frequency of heart rate variability) were associated with handling SiNPs [17]. However, cardiovascular dysfunction was not found to be associated with NMs handling in their four-year panel study [18], probably attributing to selection bias and also indicating a more focus on long-term health effect in the future evaluation of NPs-caused cardiovascular toxicity.

Atherosclerosis is the major cause of cardiovascular diseases (CVDs), and its global burden is projected to rise substantially in the next few decades, particularly in developing low- and middle-income countries [19, 20]. Existing studies have shown the role of engineered NPs exposure in the pathogenesis of atherosclerosis. Pulmonary exposure of some engineered NPs, such as single-walled carbon nanotubes (SWCNTs), TiO_2_, nickel hydroxide and indium oxide NPs could accelerate the pathological progress of atherosclerosis [21–24]. But selenium NPs were recently reported to alleviate hyperlipidemia and vascular injury [25], and also, amorphous selenium quantum dots (A-SeQDs) was confirmed to prevent atherosclerosis in vivo [26]. So far, the effects of SiNPs on the formation and progression of atherosclerotic plaque is still poorly understood. Limited studies provided the vascular injury and dysfunction caused after SiNPs inhalation, probably associated with oxidative stress and inflammatory response [27]. In contrast, studies pointed out the good biological safety of SiNPs, which could improve the efficacy of cell therapy for myocardial infarction, and was considered the best candidate for stem cell therapy in cardiac tissue [28]. Owing to controversial results and to the lack of sufficient data to clearly identify the pro-atherogenic effects of SiNPs, we aim to study the long-term influence of SiNPs on the progression of atherosclerotic plaque by using ApoE-knockout (ApoE^−/−^) mice fed a Western diet and conduct in vitro experiments for mechanism research. The current study may provide persuasive evidence for safety evaluation and risk management of SiNPs, and offer a new insight into the mechanisms underlying the adverse effects of SiNPs on cardiovascular system.

## Result

### Characterization of SiNPs

**Fig. 1:**
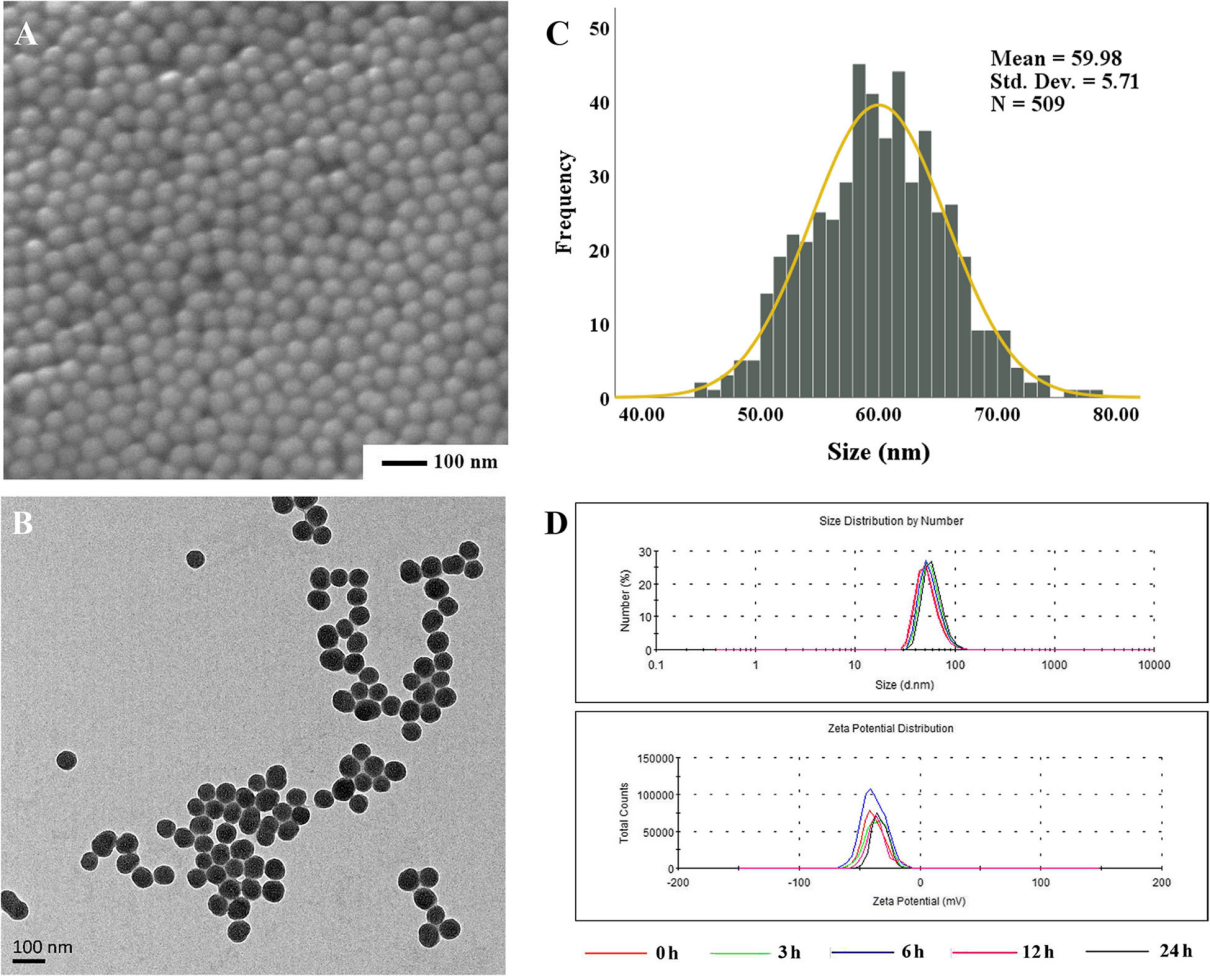
Characterization of SiNPs. Representative SEM (**a**) and TEM (**b**) images of SiNPs. Scale bar = 100 nm. **c** SiNPs are normally distributed with a mean diameter of 59.98 ± 5.71 nm. **d** The hydrodynamic size and zeta potential distribution of SiNPs in distilled water

SiNPs were spherical in shape and uniform in size as manifested by the scanning electron microscopy (SEM) and transmission electron microscopy (TEM) images (Fig. 1a, b). The particle diameter was normally distributed, with a mean value of 59.98 nm as measured by Image J software (Fig. 1c). Hydrodynamic size and Zeta potential were commonly detected as indicators of particle dispersion and stability. As a result, the hydrodynamic size and Zeta potential of SiNPs in deionized water were relatively stable at different time points (0–24 h), which were approximately 95 nm and − 35 mV, respectively (Fig. 1d and Table 1). In addition, SiNPs were sterilized and endotoxin-negative, with purity more than 99.9%.

**Table 1:** The hydrodynamic size and zeta potential of SiNPs in deionized water at different timepoints

Time (h)	Diameter (nm)	Zeta potential (mV)
0	96.23 ± 3.76	−36.40 ± 3.13
3	102.15 ± 9.08	− 36.46 ± 1.25
6	90.83 ± 3.07	− 40.30 ± 2.48
12	89.60 ± 3.65	−34.63 ± 1.15
24	95.48 ± 5.31	−33.86 ± 1.53

**Note:** Data are expressed as mean ± SD, *n* = 3

### Vascular stiffening promoted by SiNPs in ApoE^−/−^ mice

**Fig. 2:**
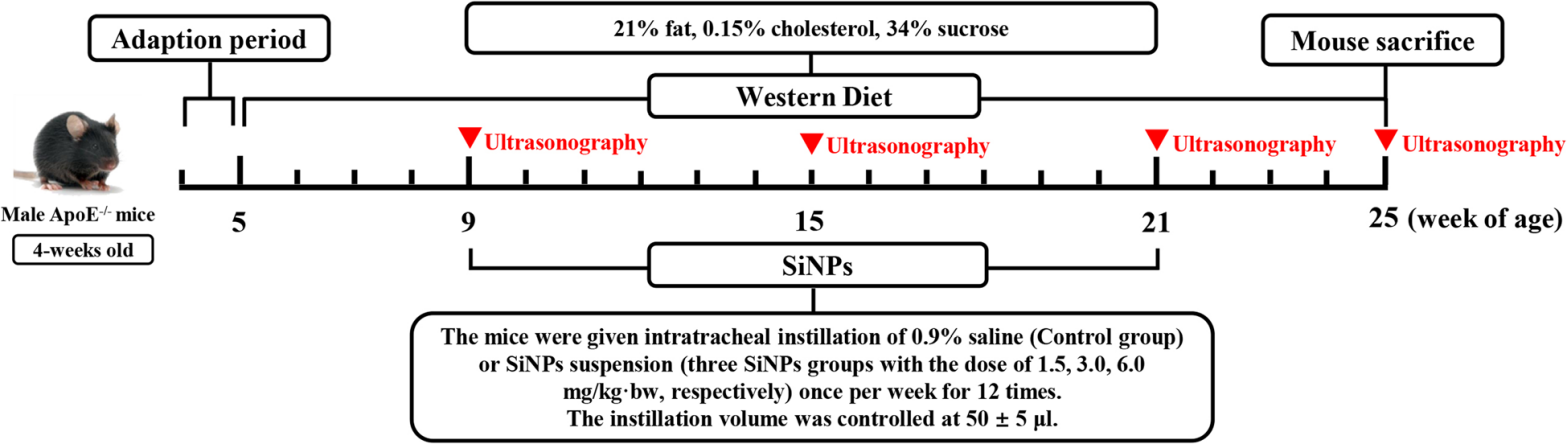
Experimental design of SiNPs promoted the atherosclerotic plaque progression in ApoE^−/−^ mice. 4-week-old male ApoE^−/−^ mice fed a Western diet were exposed to SiNPs via intratracheal instillation for 12 times, once per week, in order to investigate the influence of sub-chronic SiNPs exposure on the development of atherosclerosis

**Fig. 3:**
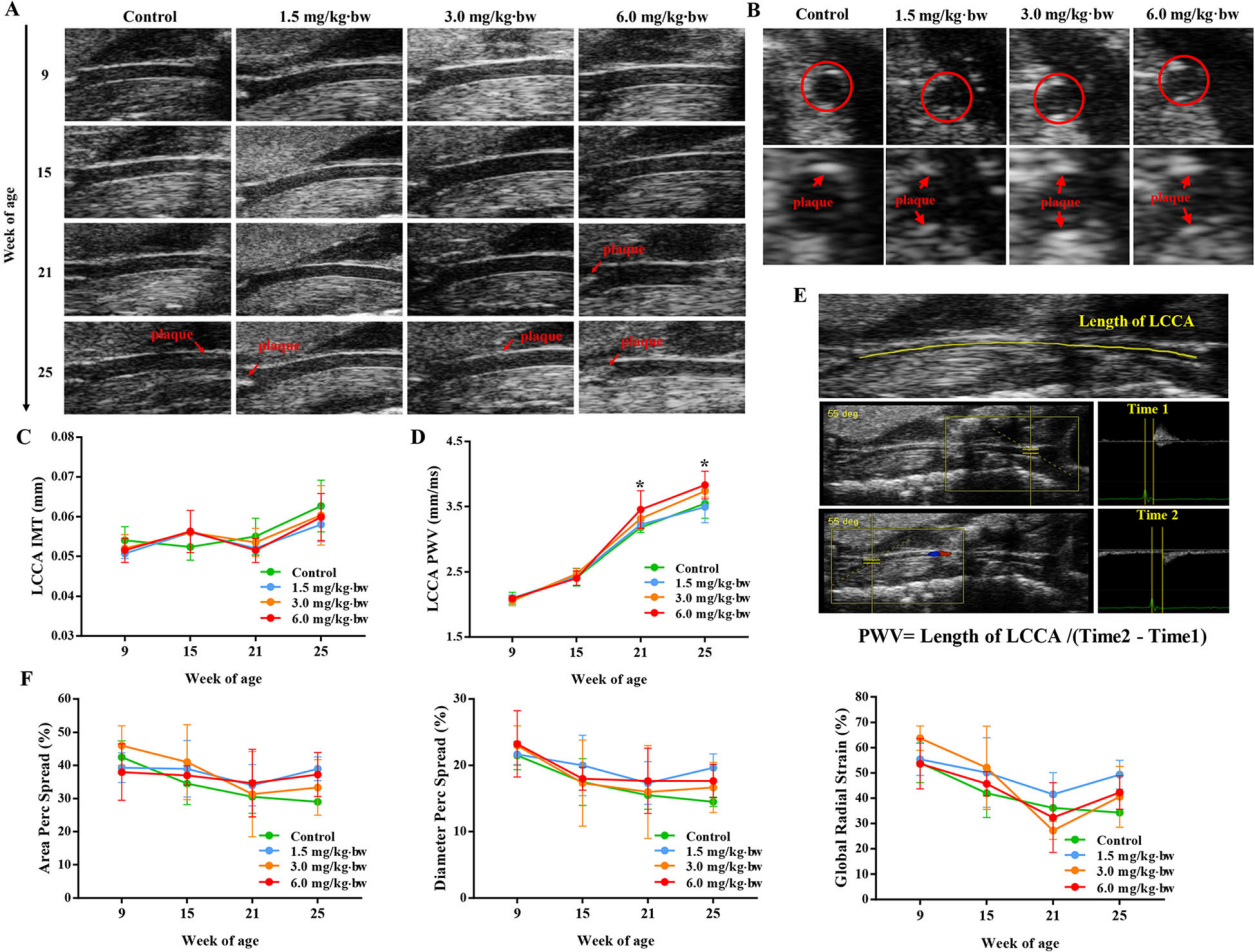
SiNPs promoted the stiffness of carotid artery in ApoE^−/−^ mice. **a** Representative B-mode ultrasound images of the LCCA (long axis) at each timepoint. **b** Representative B-mode ultrasound images of LCCA (short axis) at the end of experiment. The IMT (**c**), PWV (**d**), area percent spread, diameter percent spread and global radial strain (**f**) of the LCCA were measured at different timepoints. **e** indicates the method for detecting PWV. *p < 0.05 vs Control group at the same timepoint. Data are expressed as means ± SD, *n* = 3

The effect of SiNPs on atherosclerosis was firstly evaluated by using in vivo model, which was achieved by intratracheal instillation of SiNPs on ApoE^−/−^ mice fed a Western diet. Moreover, the ultrasound biomicroscopy (UBM), a useful tool for the non-invasive, dynamic characterization of blood vessels in animal models, was applied to monitor the vascular morphology and elasticity, and repeated measurement data was acquired. The experimental design was shown in the Fig. 2. Finally, plaques within the left common carotid artery (LCCA) were gradually formed and observed by UBM as the experiment progressed (Fig. 3a and b), and the intima-media thickness (IMT) and pulse wave velocity (PWV) in each group increased (Fig. 3c-e). The area/diameter percentage spread (changes in displacement of blood vessels during a cardiac cycle) and global radial strain were declined at different timepoint (Fig. 3f), which was consistent with the process of atherosclerosis. To be noted, PWV value of LCCA was greater in SiNPs group (6.0 mg/kg·bw) when compared to control group. As a classic indicator of vascular stiffness [29], the increased PWV value suggested SiNPs exposure could exacerbate vascular stiffening of ApoE^−/−^ mice.

### Atherosclerotic plaque progression promoted by SiNPs in ApoE^−/−^ mice

**Fig. 4:**
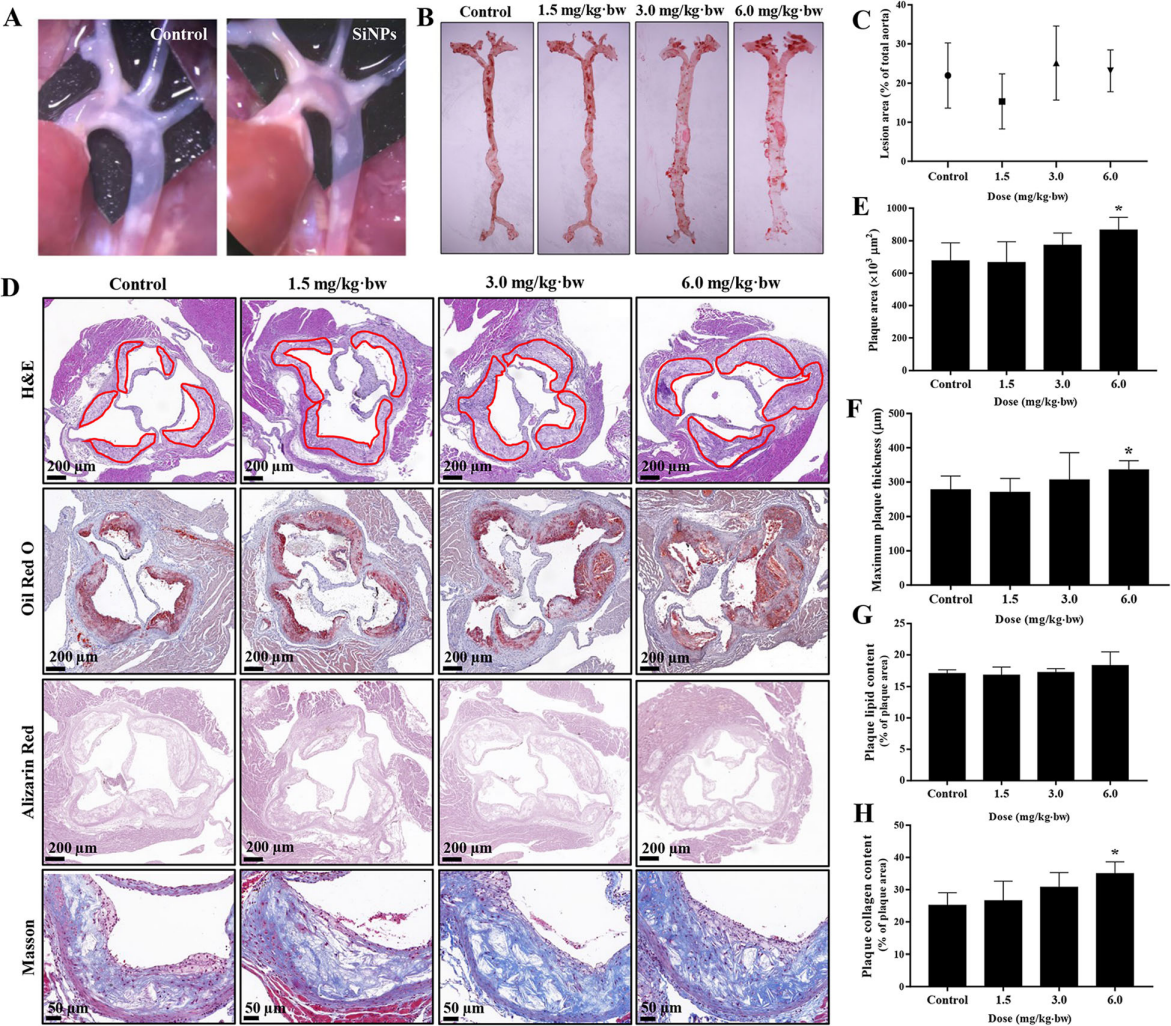
Effect of SiNPs on the progression of atherosclerosis. Representative images of a mouse aorta with atherosclerotic plaque (**a**), and the whole aorta stained by Oil red O was arranged for optimal measurement of lesion area by en-face analysis (**b**, **c**). *n* = 3. **d** Representative images of H&E, Oil Red O, Alizarin Red and Masson staining in aortic root. Scale bar = 200 μm or 50 μm. **e**, **f** The analysis of plaque area and maximum plaque thickness based on H&E and Oil Red O staining, *n* = 7–9. **g** The plaque lipid content based on Oil Red O staining, *n* = 4. **h** Plaque collagen content based on Masson staining, *n* = 4. *p < 0.05 vs Control. Data are expressed as means ± SD

**Fig. 5:**
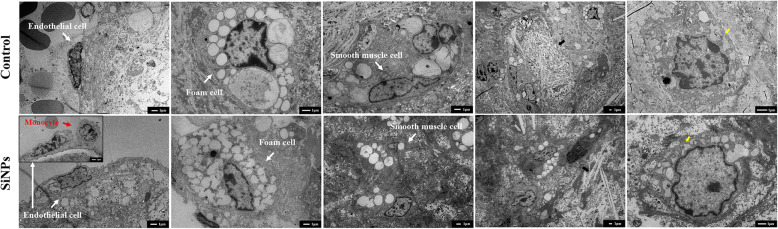
Representative TEM picture of the plaque. Foam cells, migrated smooth muscle cells, and a large quantity of cholesterol crystals and necrotic substances (black arrow) were observed in the lesions of mice in control or SiNPs group. The expansion of endoplasmic reticulum was observed within macrophages inside the lesion (yellow arrow). Scale bar = 1 μm

The distribution and progression of lesions in the aorta was assessed through en-face staining of the whole aorta and histopathological analysis of plaques in aortic root. As depicted in Fig. 4a, there were significant plaque formation in mice aorta of each group, while no obvious difference was observed on the distribution of plaque in the whole aorta after SiNPs exposure when compared to the control group (Fig. 4b and c). Further, hematoxylin and eosin (H&E), Oil-Red O staining of aortic root sections were used to quantify the plaque burden (Fig. 4d). As shown in Fig. 4e and f, the lesion area and the maximum plaque thickness were increased after SiNPs exposure (6.0 mg/kg·bw; *p* < 0.05). Oil-Red O staining revealed a large amount of lipid deposition inside the plaque and a slight increase trend of lipid content after SiNPs exposure but no significance in comparison to the control group, probably attributing to the higher plaque area in SiNPs-treated mice (Fig. 4g). Masson staining showed an increase of collagen content in mice exposed to SiNPs (Fig. 4h), and the negative staining of alizarin red indicated no significant calcification occurred inside the lesion (Fig. 4d). The ultrastructure observation by TEM provided evidence for the performance of advanced plaque lesions in ApoE^−/−^ mice, which were characterized with smooth muscle cell migration, cholesterol crystal, and large amounts of necrotic substance (Fig. 5). Meanwhile, the endothelial cells injury, monocyte adhesion and foam cell formation were observed. Of note, ER expansion was clearly seen in macrophage within plaque. In addition, the pulmonary histopathological alteration was also evaluated, characterized by the alveolar destruction and inflammatory cell infiltration in SiNPs-exposed group (supplementary material Fig. S1).

### Dyslipidemia promoted by SiNPs in ApoE^−/−^ mice

**Fig. 6:**
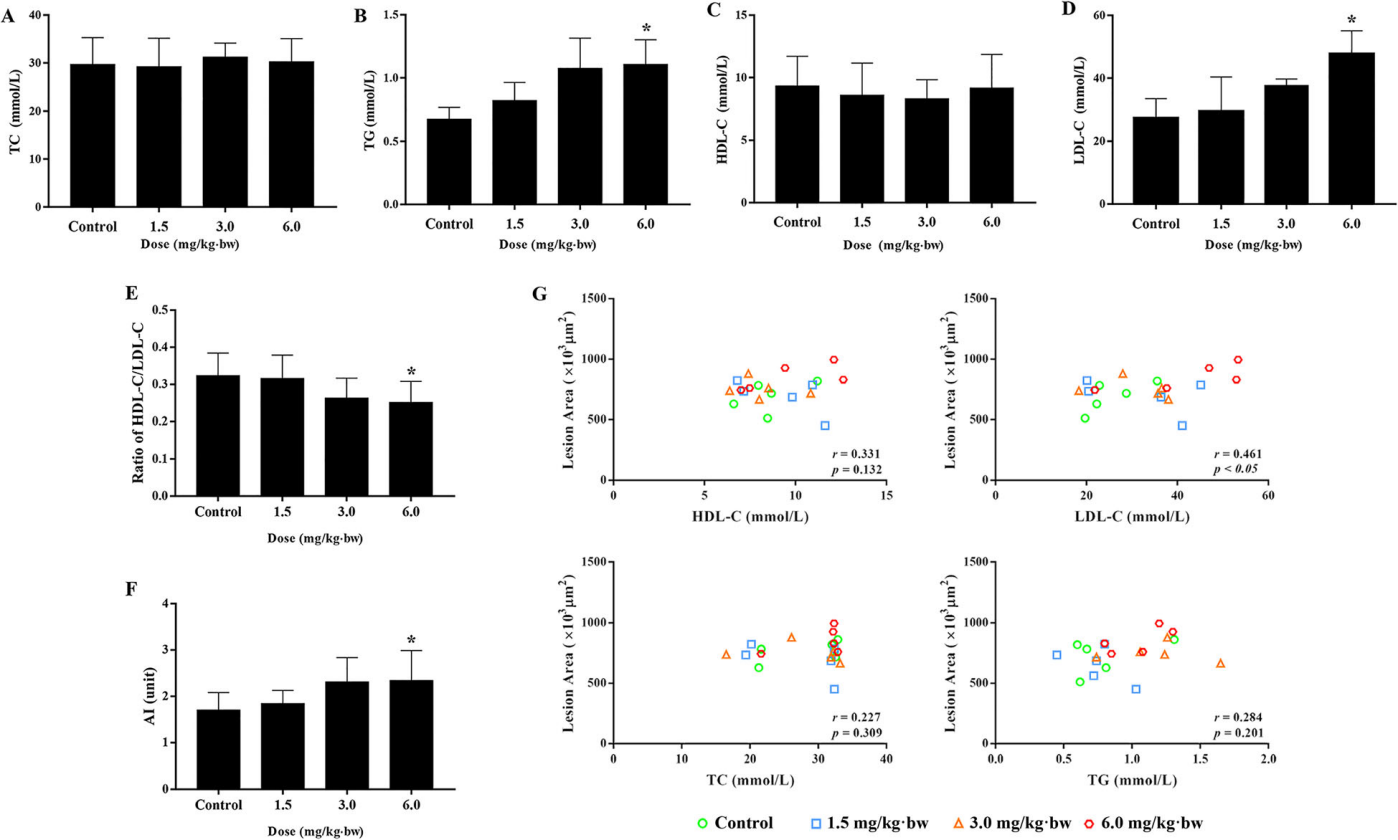
Effect of SiNPs on serum lipid profiles. The TC (**a**), TG (**b**), HDL-C (**c**), and LDL-C (**d**) were detected, and the ratio of HDL-C/LDL-C (**e**) and AI (**f**) were calculated, *n* = 6. The correlation between serum TC, TG, LDL-C, HDL-C content and aortic root plaque area was analyzed (**g**), *n* = 5. r: Correlation coefficient, *p < 0.05 vs Control. Data are expressed as means ± SD

Compared with the control group, mice in SiNPs groups had elevated total triglyceride (TG), low-density lipoprotein cholesterol (LDL-C) levels and calculated atherogenic index (AI), and declined HDL-C/LDL-C ratio, especially at the dose of 6.0 mg/kg·bw group (*p* < 0.05), in despite of no significant difference in total cholesterol (TC) and high density lipoprotein cholesterol (HDL-C) levels (Fig. 6a-f). In addition, there was a positive correlation between serum LDL-C content and plaque area in aortic root, suggesting mice with higher serum LDL-C would have a greater plaque load (Fig. 6g).

### Macrophage infiltration and ER stress triggered by SiNPs in plaque of ApoE^−/−^ mice

**Fig. 7:**
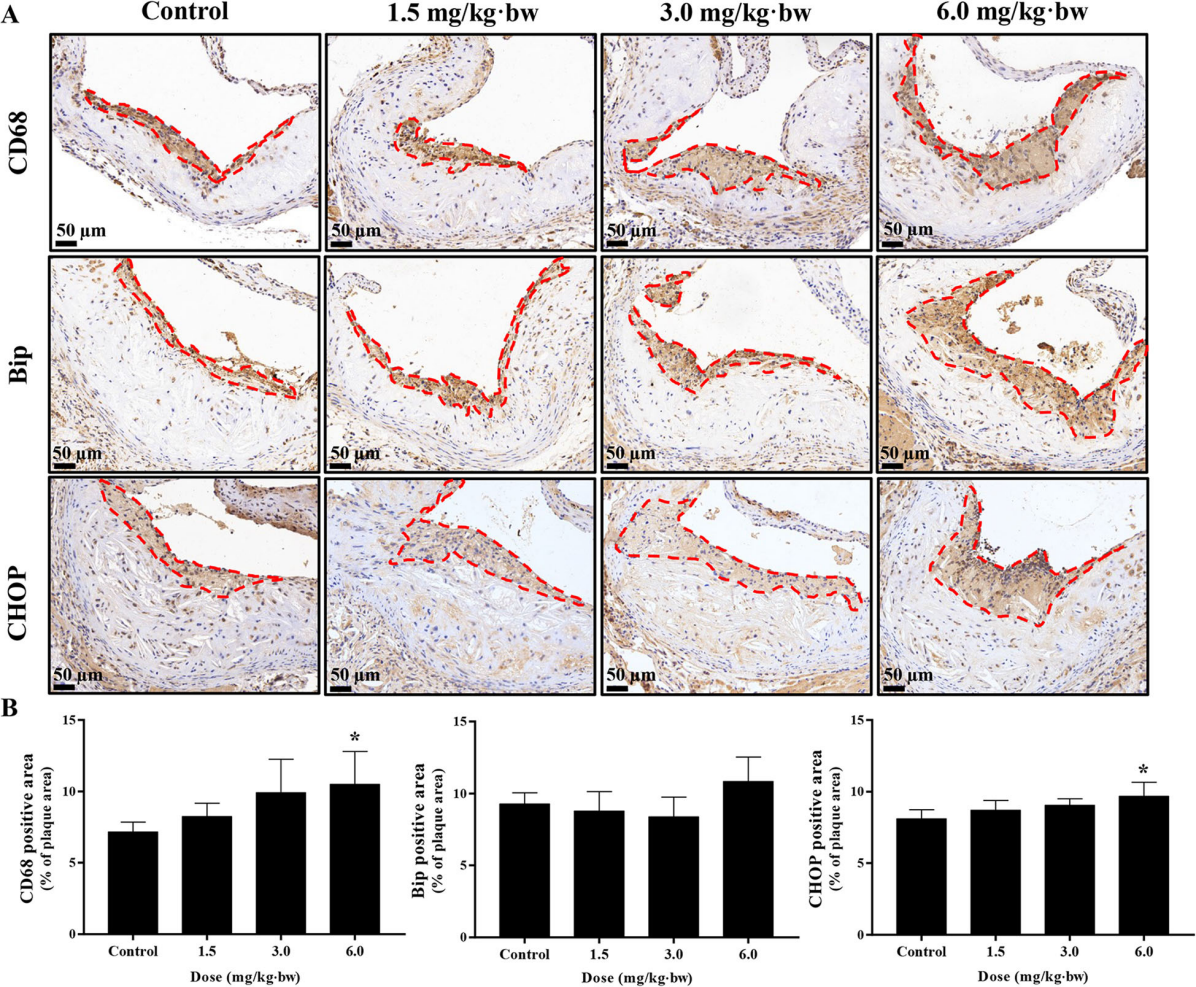
SiNPs activated ER stress in atherosclerotic lesion. **a** Representative images of immunohistochemistry staining of CD68, Bip and CHOP in aortic roots. Scale bar = 50 μm. **b** Statistical analyses of CD68-, Bip-, CHOP- positive area in aortic roots. *n* = 4. The positive area was located on the lumen side of plaque, and marked with a red dotted line. *p < 0.05 vs Control. Data are expressed as means ± SD

The macrophage infiltration and endoplasmic reticulum (ER) stress inside the lesion were assessed by immunohistochemistry. As shown in Fig. 7, the up-regulated CD68 indicated a greater macrophage infiltration within the lesion induced by SiNPs. Moreover, the elevated CHOP suggested an exacerbated ER stress within the plaque caused by SiNPs. Of note, the expressions of CD68, Bip, and CHOP were concentrated on the luminal surface of the lesion and overlapped to some extent, indirectly hinting the activation of ER stress within the macrophage.

### Particle uptake and ER stress of macrophages triggered by SiNPs in vitro

**Fig. 8:**
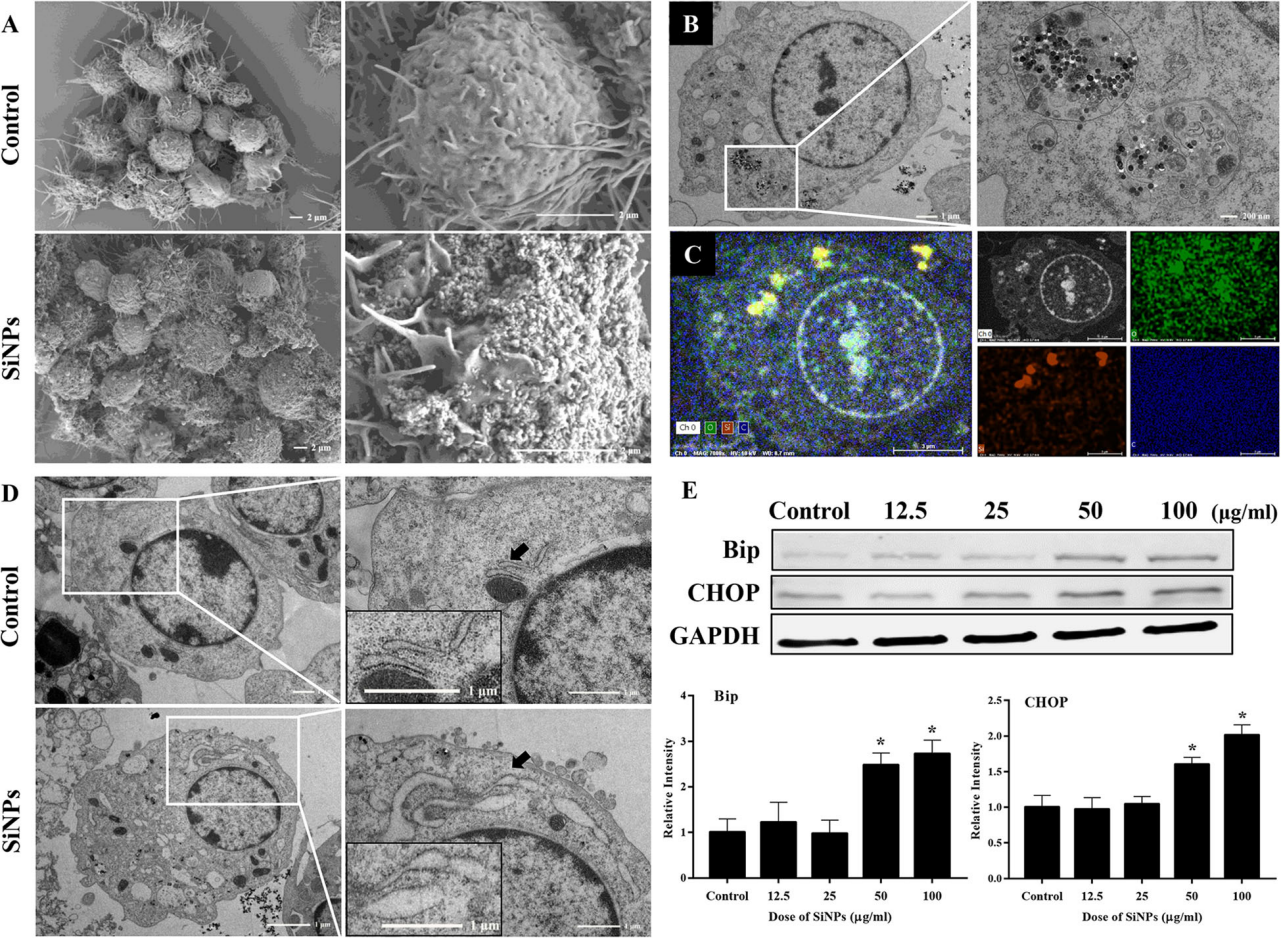
SiNPs were internalized and activated ER stress in RAW264.7 cells. The cells were treated with 50 μg/ml SiNPs for 24 h. Representative images of SEM (**a**) and TEM (**b**) respectively indicated the visualization of particle aggregates on the cellular surface, and uptake in macrophage after SiNPs treatment. Scale bar = 200 nm, 1 or 2 μm. **c** The uptake of SiNPs was further measured by energy dispersive spectrometry. In coincidence with the TEM image, silicon (red) and oxygen (green) elements were detected. Scale bar = 3 μm. **d** Representative TEM images indicated ER stress as evidenced by ER expansion and degranulation (black arrow). Scale bar = 1 μm. **e** The expressions of ER stress indicators, Bip and CHOP were detected by Western blot and the densitometric analysis was performed. *p < 0.05 vs Control. Data are expressed as means ± SD of three independent experiments

Macrophage is a major cell type involved in the onset and progression of atherosclerotic plaque, especially plays a crucial role in the regulation of inflammatory response and foam cell formation. In consideration of the observed promoting effect of SiNPs on the progression of atherosclerotic plaque in ApoE^−/−^ mice model, a murine macrophages cell line, RAW264.7 was used for the further in vitro investigations. As depicted in Fig. 8a, the protrusions and particle aggregates could be clearly observed on the SiNPs-treated cell surface. Moreover, particles were internalized and deposited in cytoplasm of macrophage, mostly in autophagic vacuoles (Fig. 8b), which were verified to be SiNPs as evidenced by the determination of silicon and oxygen elements using energy dispersive spectrometer (Fig. 8c). According to the TEM images (Fig. 8d), an expansion and degranulation of ER was evident in SiNPs-treated macrophage. In line with the TEM images, the protein expressions of Bip and CHOP were up-regulated after SiNPs exposure (Fig. 8e). All these results suggested that SiNPs could be up-taken by macrophage, and subsequently triggered ER stress.

### ER stress enhanced lipid accumulation and macrophage-derived foam cell formation by SiNPs in vitro

**Fig. 9:**
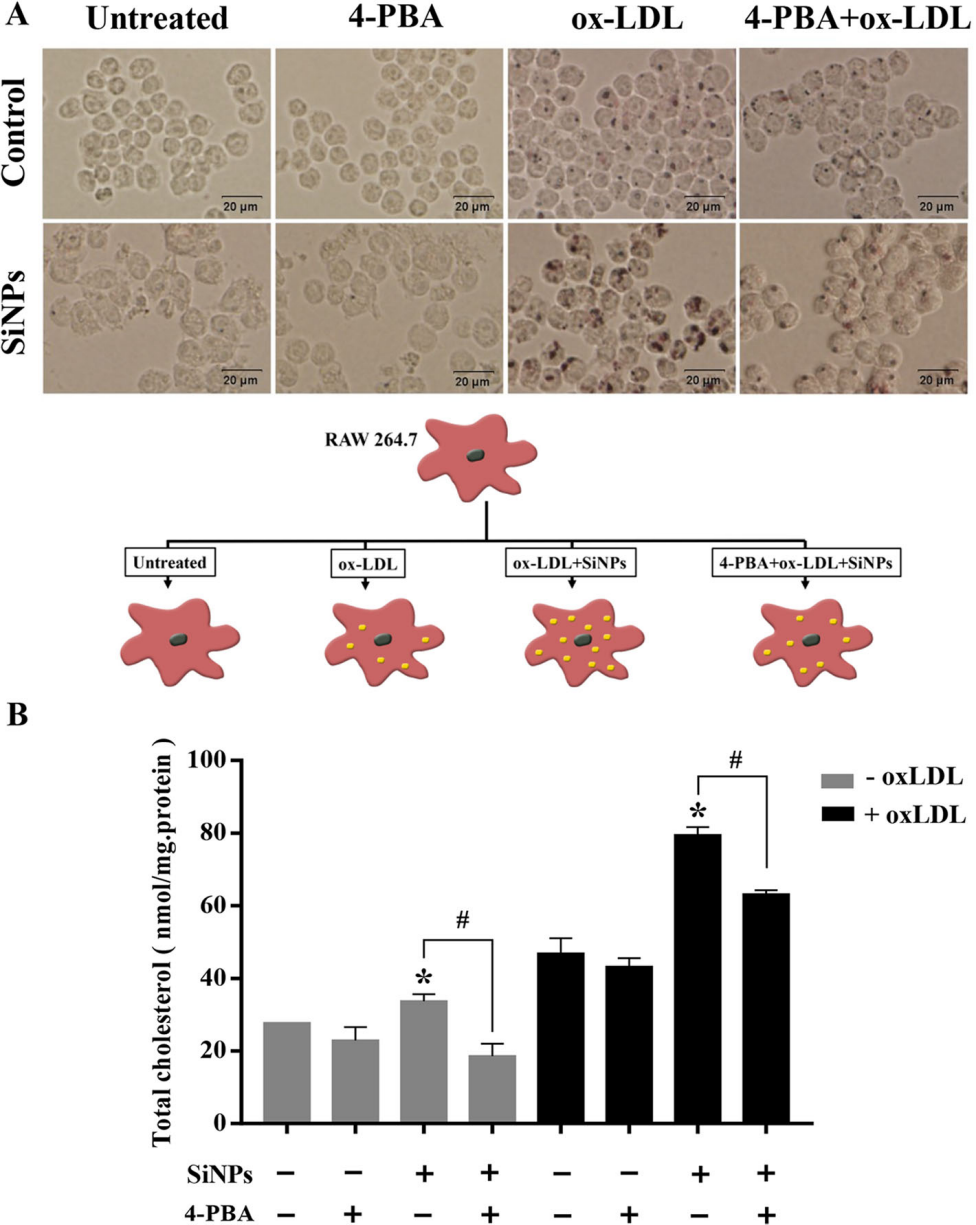
ER stress involved in the lipid accumulation induced by SiNPs in RAW264.7 cells. **a** Representative lipid droplet staining images by Oil Red O staining. Scale bar = 20 μm. **b** The intracellular total cholesterol content of RAW264.7 cells. *p < 0.05 vs Control, ^#^*p* < 0.05 vs SiNPs group with or without ox-LDL. Data are expressed as means ± SD of three independent experiments

Macrophage-derived foam cell formation is a hallmark event during the progression of atherosclerotic lesion. Oxidized LDL (oxLDL) was applied to construct a foam cell model in vitro so as to explore the role of SiNPs in the formation of macrophage-derived foam cells. As shown in Fig. 9a, Oil-Red O staining displayed SiNPs aggravated the intracellular content of lipid droplets under oxLDL cotreatment, however, 4-PBA (a classic ER stress inhibitor) pretreatment alleviated this phenomenon. Similarly, as illuminated in Fig. 9b, SiNPs treatment caused an increase in the content of intracellular TC, either with or without oxLDL cotreatment, which also could be alleviated by 4-PBA. The results validated that SiNPs promoted lipid accumulation through ER stress-mediated way, consequently contributing to the foam cell formation, even plaque progression.

### ER stress-mediated CD36 upregulation involved in lipid accumulation in macrophage by SiNPs

**Fig. 10:**
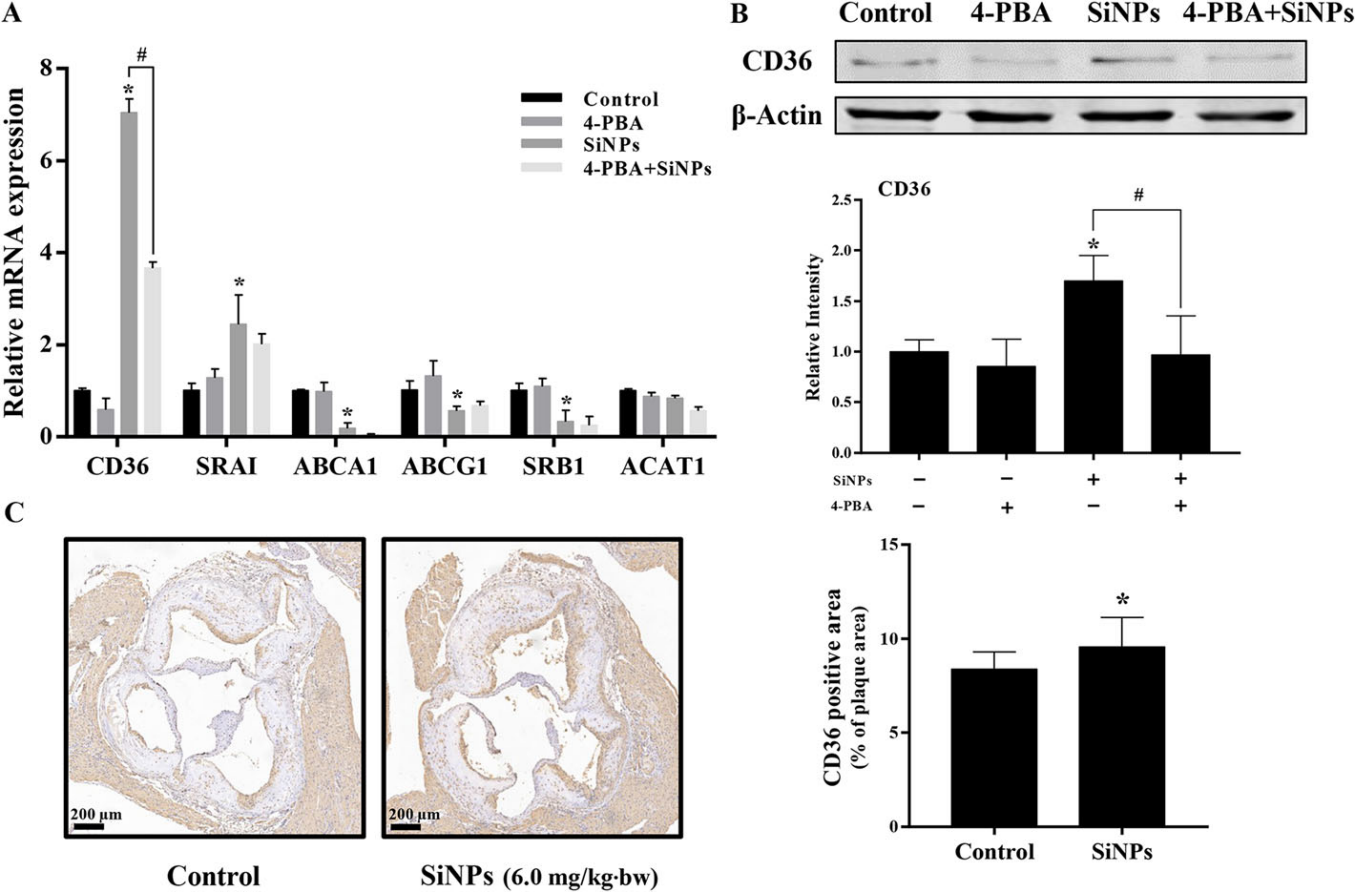
ER stress-mediated the up-regulated CD36 expression attributing to the lipid accumulation induced by SiNPs in RAW264.7 cells. **a** The relative mRNA expressions of factors involved in cholesterol influx/efflux. **b** The CD36 protein expression of RAW264.7 cells. In consistent with the mRNA level, the up-regulated CD36 expression induced by SiNPs was greatly alleviated by 4-PBA pretreatment. Also, the CD36 expression in the plaque of SiNPs-exposed aortic root was up-regualted (**c**, *n* = 4). *p < 0.05 vs Control, ^#^*p* < 0.05 vs SiNPs group. Data are expressed as means ± SD of three independent experiments

The intracellular lipid homeostasis in macrophage was precisely regulated by lipid influx and efflux. Thus, we further detected the expressions of factors controlling the cholesterol uptake (CD36 and SRA1) and its efflux (ABCA1, ABCG1, and SRB1) in RAW264.7 cells. Real-time PCR results showed that SiNPs exposure caused the up-regulated mRNA expressions of CD36 and SRA1, while down-regulated mRNA expressions in ABCA1, ABCG1, and SRB1. In addition, SiNPs had no influence on the mRNA level of ACAT1, a critical regulator to re-esterify excessive free cholesterol to cholesterol ester to store in lipid droplets [30]. Further, 4-PBA was applied to validate the role of ER stress in the regulation of lipid homeostasis. Results validated that the up-regulation of CD36 was positively regulated by ER stress, owing to a marked decline of CD36 in 4-PBA combined SiNPs group when compared to the SiNPs group (Fig. 10a). Moreover, such phenomenon was also verified in protein expression (Fig. 10b), indicating that the induction of ER stress by SiNPs could simulate CD36 expression, exacerbating lipid uptake and accumulation in macrophage. In addition, the expression of CD36 in the lesions of SiNPs-treated mice was also elevated according to the immunohistochemical analysis (Fig. 10c).

## Discussion

With the mass production and application of SiNPs, its biological safety issues, especially on the health effects of nano-handling workers need to be considered. Atherosclerotic related CVDs is the leading cause of mortality worldwide. So far, there is still no conclusive information on the pro-atherogenic potential of occupational SiNPs exposure. To the best of our knowledge, this is the first in vivo study to confirm SiNPs exposure could affect the progression of atherosclerosis. We firstly administered Western diet-fed ApoE^−/−^ mice with SiNPs through intratracheal instillation for 12 weeks by mimicking occupational scenario, and UBM was applied to monitor the development and progression of atherosclerosis. Currently, only few studies have reported the vascular effects of NPs through Doppler ultrasound trace [31]. As a result, the data indicated the atherosclerosis model was well established, and SiNPs exposure reduced arterial elasticity, aggravated the arterial stiffness. A large number of clinical studies have demonstrated an association between arterial stiffness and atherosclerotic burden [32]. PWV has also been found to have a positive association with IMT and atherosclerotic plaque formation, which has predictive value for the diagnosis of CVDs [33]. Besides, arterial stiffness was also reported to be positively correlated with reactive oxygen species (ROS) and the followed oxidative stress [34], which was commonly regarded as a major attributor of the adverse effects caused by SiNPs [35].

On the basis of a clearly observed plaque and an increased PWV value in SiNPs-treated mice, the experiment was terminated and atherosclerotic burden was assessed by histopathological staining. In despite of no difference on the plaque distribution in the whole aorta, SiNPs exposure triggered a greater plaque burden in ApoE^−/−^ mice as indicated by the histological analysis of aortic root. According to the American Heart Association’s definition of human atherosclerotic stages [36, 37], the plaque at the termination of experiment was progressed into advanced lesion with stage IV, characterized with a lipid core and complex composition inside the plaque, e.g. migrating smooth muscle cells, cholesterol crystals, and necrotic substances, but yet no obvious fibrous cap formed. Previously, SiNPs was confirmed to induce endothelial injury [35, 38–40] and promote the recruitment of monocytes to injured endothelial cells [41], and foam cell formation at the early stage of atherosclerosis [42]. Moreover, a discontinued or fragmented intimal surface was induced by repeated pulmonary exposure of SiNPs, accompanied with endothelial apoptosis [27]. In addition, the ability to induce thrombosis formation may also attribute to the pro-atherogenic potential of SiNPs [31]. However, the underlying mechanisms by which SiNPs influenced atherogenesis still remains largely unknown.

Lipid is one of the most important stimuli initiating atherogenesis and the plasma lipoproteins are involved in foam cell formation and inflammatory regulation within plaques [43–45]. The abnormal plasma lipoprotein level is usually considered as the feature of atherosclerosis. Repeated intravenous administration of SiNPs was reported to disturb hepatic lipid metabolism and trigger hyperlipidemia in mice [46]. Similarly, dys- or hyper-lipidemia was induced after a long-term exposure of Zinc oxide (ZnO) NPs via intratracheally instillation in rat, or of TiO_2_ NPs in ICR or ApoE^−/−^ mice, ultimately contributing to the initiation and progression of atherosclerosis [24, 47, 48]. In particular, a cross-sectional study also found that occupational exposure to TiO_2_ NPs affected lipid metabolism, as evidenced by a higher level of serum LDL when compared to the normal physiologic range for LDL in adults in China [14]. In agreement with these findings, we revealed the sub-chronic SiNPs treatment via intratracheal instillation also aggravated the hyperlipidemia of ApoE^−/−^ mice, characterized with the increased serum levels of TG and LDL-C. To be noted, the increase in serum LDL-C content was positively correlated with the plaque area in aortic root. Meanwhile, the AI index was increased, whilst HDL-C/LDL-C ratio was decreased in SiNPs group when compared with the control group, hinting the dyslipidemia caused by SiNPs may contribute to the progress of atherosclerotic lesions [49, 50]. Coincidently, population studies have shown a highly consistent, positive correlation between blood LDL-C level and atherosclerotic CVD risk in humans [51]. Besides, hypertriglyceridemia is causally associated with increased atherosclerosis risk [44]. Intriguingly, either the repeated exposure to SWCNT or long-term (over 5 months) exposure of nano-Ni was reported to exacerbate plaque development in ApoE^−/−^ mice, but no alteration of lipid profile [22, 24]. It might explain other mechanisms also contributing to atherogenesis, such as systemic oxidative stress, inflammation [52].

ER stress, also known as unfold protein response (UPR), plays a crucial role in the pathogenesis of a series of cardiovascular disorders, including atherosclerosis, ischemia [53]. ER is a major site for protein folding and calcium reservoir. Numerous studies suggested ER as a potential target for NPs [38, 54–58]. The accumulation of misfolded or unfolded proteins led to ER stress, which was proposed as the mechanism responsible for NPs-induced toxicity [55]. ER stress occurs at all stages of atherogenesis [59], and plaques with higher levels of ER stress show faster plaque progression [60]. The induction of ER-associated UPR events by NPs was pointed out either in vitro or in vivo [38, 61]. In particular, other than oxidative stress, ER stress was reported to mediate the SiNPs-caused vascular injury in rats [27]. To be noted, ER stress mediates cell apoptosis and lipid metabolism [62], and interacts with oxidative stress and autophagy, which are closely related to atherosclerosis [63, 64]. Therefore, ER stress is likely to be the key factor for NPs and their cardiovascular effects. However, most existing studies on NPs focus on the ER stress-mediated apoptosis process [61], and only a few studies found that ER stress induced by NPs may be involved in regulating cellular lipid metabolism [42, 65].

Macrophage-derived foam cell is the crucial determinant of the initiation and progression of atherosclerosis lesion [66], contributing to plaque instability and rupture [67]. In agreement with previous studies [42], ER stress was significantly induced after SiNPs exposure, as evidenced by the expansion and degranulation of ER, as well as greatly up-regulated Bip and CHOP expressions. More importantly, ER stress inhibition largely alleviated the lipid accumulation induced by SiNPs in macrophage as assessed by the Oil-Red O staining and intracellular cholesterol measurement. It is worth noting that some ER stress genes are involved in lipid metabolism, which fuel atherogenesis. For instance, CHOP is crucial for lipid synthesis, and its expression would result in lipid accumulation [68]. The cellular lipid homeostasis is highly, precisely regulated by lipid influx and efflux. Upon oxLDL and SiNPs co-exposure, dysregulated expressions of lipid influx/efflux genes were detected in macrophage [42], probably mediated by ER stress signaling. Further, Long et al. revealed the lipid accumulation in macrophages was attributed to the modulation of ER stress leading to the upregulation of scavenger receptors, including CD36 and SRA1 [65]. Apart from CD36, ER stress has also been reported to correlate with reduced ABCA1 level in macrophage, a key regulator for lipid efflux [69, 70]. However, our data confirmed only CD36 was dependent on ER stress induced by SiNPs, leading to lipid accumulation and foam cell formation, which ultimately contributing to atherosclerosis. CD36 is a membrane glycoprotein that belongs to the class B scavenger receptor family, and is known to be involved in lipid metabolism as well as atherosclerosis development [71]. In line with our finding, CD36 was reported to participate lipid accumulation caused by other NMs (e.g., MWCNTs, ZnO NPs) [65, 72]. A series of studies have proven ER stress modulated lipid influx via CD36 [73–75]. Lipid could be trafficked by CD36 to ER, and meanwhile, the accumulation of toxic lipids in macrophages would result in a prolonged ER stress [76]. However, the detailed molecular mechanisms by which ER stress regulate CD36 need to address in future studies. Besides for oxLDL uptake, CD36 can also affect atherosclerosis by combining with various ligands, specifically in regulating inflammation, endothelial dysfunction, macrophage migration, and hyperlipidemia etc. [77]. Studies have confirmed CD36 plays a critical role in vascular dysfunction caused by NP exposure [78, 79]. CD36 was also reported to activate NLRP3 inflammasome in response to atmospheric particulate matter exposure [80], as well as modulating lipid accumulation in macrophage [81], ultimately contributing to the progression of atherosclerosis.

## Conclusions

In summary, this is the first study to directly address the acceleratory effect of SiNPs in atherosclerotic plaque progression by using ApoE^−/−^ mice. And also, it is novel in the utilization of ultrasonic technique to non-invasive, dynamic assess the vascular function and plaque formation. Consequently, the current study demonstrated an increased plaque size, macrophage infiltration and ER stress in the aortic root after long-term SiNPs exposure via intratracheal instillation, accompanied by the aggravated hyperlipidemia and artery stiffness in plaque. The phenomena mentioned above was significantly manifested in the SiNPs group with a higher dose (6.0 mg/kg·bw), which was closely associated with the exposure mode (dose, time, interval, etc.). Specific details about the dose selection and conversion can be seen in Method part. Moreover, a comprehensive molecular mechanism related to the promotion of atherosclerotic progression by SiNPs was provided. That was, ER stress-mediated up-regulated CD36 expression was validated to be a major contributor of SiNPs to promote foam cell formation and ultimate plaque progression. Overall, our results provide new insight into the cardiovascular toxicological effects of SiNPs, and inhibition of ER stress might be a promising approach to alleviate NPs-induced vascular lesion. Besides SiNPs, other NMs, e.g. carbon nanotubes, TiO_2_ NPs, ZnO NPs, were found to have pro-atherogenic potential, ultimately leading to the onset and development of CVDs. Hence, NPs exposure may aggravate the cardiovascular risk of occupational population, and more efforts should be made for ensuring occupational safety, and also for a reasonable and safer application of nanoproducts.

## Methods

### Nanoparticles preparation and characterization

The amorphous SiNPs used in the experiments were prepared by the Stöber method as previously described [35]. The particle shape and size was observed by scanning electron microscopy (SEM; Hitachi S-4800, Japan) and transmission electron microscopy (TEM; JEM2100, Japan). Based on the TEM results, the particle size distribution was analyzed through Image J software. The hydrodynamic size and Zeta potential of SiNPs in deionized water were measured by Zetasizer (Malvern Nano-ZS90, UK). Moreover, an inductively coupled plasma atomic emission spectrometry (ICP-AES; Agilent 720, USA) was used for the purity detection of the synthesized SiNPs, and a gel-clot limulus amebocyte lysate (LAL) assay kit (Bokang, Zhanjiang, China) for endotoxin measurement. In addition, the stock suspension of SiNPs were firstly dispersed by a sonicator (160 W, 20 kHz, 5 min; Bioruptor UCD-200, Belgium), and then diluted by the corresponding exposure media, 0.9% saline (in vivo test) or DMEM (in vitro test).

### Animal studies

ApoE^−/−^ mice at the age of 1–2 months is commonly used as the animal model for spontaneous atherosclerosis [82]. Male ApoE^−/−^ mice (age, four-week; weight, 18–22 g) were obtained from the Experimental Animal Center of Capital Medical University (Beijing, China) to assess the long-term effect of SiNPs in the development of atherosclerosis. All mice were housed in sterilized filter-topped cages with free access to food and water, and maintained in a specific pathogen-free facility with a constant humidity (50 ± 5%), temperature (24 ± 1 °C) at 12/12-h light/dark cycle. After 1 week of acclimation, all the mice were supplied with a Western diet (21% fat, 0.15% cholesterol, 34% sucrose) for a rapid establishment of the murine atherosclerosis model. According to the development stage of atherosclerosis in ApoE^−/−^ mice fed with high-fat diet [83], the SiNPs exposure began when mice was 9-week old. The mice were randomly divided into four groups, which were control group and three SiNPs groups at a dose of 1.5, 3.0, or 6.0 mg/kg·bw, respectively.

The applied dose of SiNPs was in reference to an inhalation study in mice [84], in which the doses of SiNPs were evaluated based on the real workplace exposure scenarios. It was reported the occupational exposure level of SiNPs ranged from 1.0–27.6 mg/m^3^ [85]. In considering the lack of a recommended exposure limit for amorphous SiNPs, the permissible concentration-time weighted average (PC-TWA) of amorphous silica dioxide (SiO_2_), 6 mg/m^3^ was used. Therefore, a worker (60 kg) exposed to a concentration of 6 mg/m^3^ SiNPs for 8 h (1 workday) without proper protection would result in an approximate pulmonary dose of 0.44 mg/kg·bw (assuming human under a light exercise condition in workplace with breathing frequency 20 breaths/min, 1024 mL/breath, and pulmonary deposition fraction for 60 nm particles of 0.45 in human) [86, 87]. According to the equivalent conversion coefficient of the dose per kilogram of body weight in experiment animals and human [88], the dosage is equivalent to 5.45 mg/kg·bw in mice. Thus, we set the highest dose as 6.0 mg/kg·bw. In addition, Inoue et al. mentioned the number of NPs in ambient air ranged from 2 × 10^4^ to 2 × 10^5^/cm^3^, with mass concentrations of > 50 μg/m^3^ near major highways [89]. Here, the actual lung exposure dose of SiNPs (1.5, 3.0 or 6.0 mg/kg·bw) was about 40, 80, 160 μg/mouse/week (based on mice weighting 26–29 μg during SiNPs treatment), respectively. According to a previous study [48], the inhalation dose of a mouse is about 5 μg after a one-week exposure at the daily concentration of 50 μg/m^3^ near major highways (consuming the inhalation rate for mice is 0.052 m^3^/day, and the mice pulmonary deposition fraction for 60 nm particles of 0.25) [87]. Thereby, the applied dose (1.5, 3.0 or 6.0 mg/kg·bw) in this study was correspondingly 8, 16, or 32 times to the airborne exposure level of NPs.

Mice in SiNPs groups were administered SiNPs suspension through intratracheally instillation, once in every 7 days and 12 times in total, whereas the control mice were instilled with 0.9% saline instead. The volume of intratracheal instillation was controlled to be 50 ± 5 μl. Furthermore, the UBM of three mice per group was performed during the experiment. In addition, the body weight and food intake of mice were monitored and weighed weekly (see details in the supplementary Fig. S2). The experiment was terminated at 1 month after the last SiNPs exposure in order to observe an irreversible effect caused by SiNPs exposure. At the termination of experiment, mice were fasted overnight, blood and aortas were harvested. Serum was extracted from blood and stored at − 80 °C until analyzed. All the animal experimentation was performed following the National Guidelines for Animal Care and Use, and approved by the Committee of Laboratory Animal Care and Use in Capital Medical University (Ethical number, AEEI-2018-002).

### Ultrasound biomicroscopy

An ultra-high resolution color doppler ultrasound system (Vevo 2100, FUJIFILM Visualsonics, USA) equipped with MS 400/550D mechanical transducers were used, and the ultrasound imaging parameters of LCCA were measured. During the experiment, mice were anaesthetized with isoflurane gas resulting in a heart rate of approximately 500 beats/min, and the hair from the anterior chest wall was carefully shaved, and warm ultrasound transmission gel was liberally applied to ensure optimal image quality. The IMT and PWV were obtained using VEVO LAB software. The EKV two-dimensional dynamic image was analyzed by VEVO VASC software, and indicators representing vascular compliance (diameter/area percentage spread and global radial strain) were obtained. On the basis of a previous description [90], IMT was measured with the vascular lumen-intimal interface selected as the internal measurement site and the media adventitial interface as the external limit. PWV was calculated by the following formula: Length of LCCA/Time of blood flowing through the LCCA. All measurements were repeated three times. All the images were analyzed by another operator blinded to the identities of the animals.

### Lipid profiles analysis

Blood samples were collected and centrifuged at 3000 rpm, 4 °C for 10 min. The contents of TC, TG, HDL-C, and LDL-C in mice serum were measured by an automatic biochemical analyzer (HITACHI 7180) combining commercial kits (Jiancheng, Nanjing, China). The ratio of HDL-C/LDL-C was calculated, as well as AI according to the formula: AI = (TC - HDL-C)/HDL-C [49].

### Histopathological examination

For lesions throughout the aorta, the whole aortas of three mice per group were separated, cut longitudinally after removing excess adipose tissue, and stained with Oil-Red O staining (Solarbio, Beijing, China) for 10 min. Afterwards, the stained aortas were placed in 75% alcohol until the artery wall without lesions was cleaned. Images were captured and analyzed by Image J software. Furthermore, for the lesion at the aortic root, the entire aortic root was immersed in 4% paraformaldehyde for 24 h, embedded into paraffin or optimal cutting temperature (OCT) for histological examination. The cross-sections of the aortic root were stained with H&E, Oil-Red O, Masson and Alizarin Red for the quantification of plaque area, lipid and collagen content, and aortic calcification. It is worth noting that the regional error in lesion size was avoided by acquiring of the sequential cross-sections throughout the entire aortic root as previously described [91]. Ultimately, the largest lesion area was selected for the comparative analysis of plaque (Supplementary material Fig. S3). All slides were scanned with Pannoramic SCAN system (3DHISTECH, Hungary), and measured with CaseViewer software (3DHISTECH, Hungary) or by Image J software. The quantification of each morphological parameter was performed by one investigator blinded for the treatment, and reviewed by certified veterinary pathologists.

### Immunohistochemical staining

Immunohistochemistry was performed in the paraffin-embedded artery root to determine the expressions of CD68 (a macrophage marker), CD36 (a principal contributor to cholesterol uptake), Bip and CHOP (biomarkers for ER stress) in situ. Briefly, the dehydrated paraffin sections were immersed in 1 mM EDTA (pH = 9) for antigen retrieval, and incubated with 3% hydrogen peroxide to abolish endogenous peroxidase. The sections were incubated with the primary antibody for CD68 (ab125212, Abcam, UK), CD36 (18,836, Proteintech, USA), Bip (#3177, CST, USA), or CHOP (15,204, Proteintech, USA) overnight at 4 °C, and then incubated with the corresponding secondary antibody and stained with 3,3′-diaminobenzidine (DAB). These primary antibodies were diluted with 5% BSA solution at a ratio of 1: 200. Moreover, the nucleus was stained with hematoxylin. Finally, the percentage of positive-staining area in the whole plaque of aortic root was analyzed using the Image J software. All analyses were performed by one investigator blinded for the treatment.

### TEM observation of lesions

The ultrastructure of lesion was observed by using TEM (JEM2100; JEOL, Japan). In brief, the first branch of aorta was fixed by 2.5% glutaraldehyde overnight, rinsed with 0.1 M phosphate buffer, and postfixed with osmic acid for 2 h. After being dehydrated in ethanol with concentration gradients and acetone, the sample was embedded in epoxy resin. Ultimately, the ultrathin sections (50 nm) were obtained and imaged under TEM.

### Cell culture and treatment

Mouse macrophage cell line, RAW264.7 cells were cultured in DMEM (ThermoFisher, USA) with 10% fetal bovine serum (FBS; ThermoFisher, USA) at 37 °C in a 5% CO_2_ incubator. SiNPs were diluted by DMEM to appropriate concentrations, and an ER stress inhibitor, 4-phenylbutyric acid (4-PBA; Selleck, USA) was applied (3 mM, 6 h). The dosage of SiNPs was set according to the cell viability analysis by using MTT assay. Since a significant acute toxicity (24 h) was seen in SiNPs-treated group at a concentration of 50 μg/ml, while simultaneously with cell viability > 70%, the exposure mode of SiNPs (50 μg/ml, 24 h) was used in the subsequent in vitro experiments. Similarly, the application of 4-PBA was set up through MTT assay and also verified as evidenced by an efficient inhibition on the up-regulated expressions of Bip and CHOP induced by SiNPs. See details in the supplementary Fig. S4. After SiNPs with or without 4-PBA treatment, cells were harvested for the following measurement.

### Cellular morphology observation and particle internalization analysis

After 50 μg/ml SiNPs treatment for 24 h, the cellular morphology and alterations of cellular ultrastructure were observed by SEM (S-4800, Hitachi, Japan), and TEM (JEM2100, JEOL, Japan). Based on the TEM image, the particle uptake and internalization were verified by energy dispersive spectrometry (EDS; Bruker-XFlash6/60, Germany).

### Intracellular lipid measurement

Intracellular lipid droplets were determined by Oil-Red O staining as previously described [42]. In brief, cells were fixed with 4% paraformaldehyde, and stained with Oil-Red O working solution for 30 min after assimilation of 60% isopropanol. The excess dye was washed away with 60% isopropanol and the cells were observed under an Olympus IX81 microscope (Tokyo, Japan). Also, the intracellular content of total cholesterol (TC) was measured by a total cholesterol assay kit (Applygen, Beijing, China) according to the manufacturer’s protocol. Ultimately, the intracellular TC content was calibrated using protein mass.

### Quantitative real-time RT-PCR

The total cellular RNA was extracted by using a RNAsimple Total RNA kit (Tiangen, Beijing, China), and reversely transcribed to cDNA using a PrimeScript™ RT reagent kit (TaKaRa, Japan). The quantitative PCR was performed by using the SYBR Premix Ex TaqTM II (Takara, Japan) in a real-time PCR machine (Bio-Rad, USA). The relative expression in mRNA levels of lipid transport (CD36, SRA1, ABCA1, ABCG1 and SRBI) and esterification (ACAT1), and also ER stress indicators (Bip and CHOP) were quantified. Each experiment was conducted in triplicate with β-actin as the internal standard. Primers used for quantitative PCR analysis were listed in the supplementary file (Table S1).

### Western blot assay

The whole cellular protein was extracted by a Protein Rapid Extraction kit (KeyGEN, China), and quantified by BCA protein assay (Dingguo, China). After denaturation, protein lysate was separated with SDS-PAGE, and transferred to a nitrocellulose membrane (Pall, Germany). The membrane was blocked with Tris-buffered saline (TBS) solution containing 5% skim milk powder for 1 h at room temperature. After wash three times with TBST (TBS with 0.05% Tween-20), membrane was incubated with the primary antibody diluted by TBST solution (1: 1000), including Bip (#3177, CST, USA), CHOP (#2895, CST, USA), CD36 (ab64014, Abcam, UK), GAPDH (#5174, CST, USA) and β-actin (66,009, Proteintech, USA) overnight. After three-time wash with TBST, membrane was incubated with the corresponding fluorescent secondary antibody (LI-COR, Gene Company Limited, Hong Kong) for 1 h at room temperature, and ultimately detected using Odyssey® CLx imaging system (Gene Company Limited, Hong Kong). At least three independent experiments were performed. The relative expression level of protein was analyzed by Image Studio™ quantification software (Gene Company Limited, Hong Kong) with β-actin or GAPDH as internal control, and normalized to the control group.

### Statistical analysis

Data were expressed as mean ± standard deviation (SD). Significant differences in ultrasound data were analyzed by ANOVA of repeated measurement data. The t-test of independent samples was used to analyze the significant difference of the intracellular cholesterol content and lipid influx/efflux factor expression in RAW264.7 cells, as well as CD36 expression in mouse plaque lesions. Significant differences in the remaining data were analyzed by one-way ANOVA. The LSD test was selected for post hoc test of homogeneous data, whereas Dunnett’s T3 test for the post hoc test of heterogeneous data. A two-tailed Pearson correlation test was applied to determine the correlation between the lesion areas in aortic root and serum lipid levels. All data were analyzed by SPSS 20.0 software, and *p* value < 0.05 indicates statistical significance.

## Supplementary information

**Additional file 1 Fig. S1**. Histopathology alterations of lung tissues after intratracheal instillation exposure to SiNPs in ApoE^−/−^ mice. *n* = 4 per group. **Fig. S2.** The body weight and food intake trends of mice. The body weight and food intake of the mice was measured weekly during the experiment. Data are expressed as means ± SD, *n* = 9. **Fig. S3**. Method for analyzing plaque area in aortic root. Regional errors in lesion size were avoided by acquiring sequential throughout of the entire aortic root. The lesion with the largest area was used for statistical analysis. **Fig. S4** 4-PBA inhibited the ER stress induced by SiNPs in RAW267.4 cells. (A) Cell viability was determined by MTT assay after 24 h-treatment with SiNPs. (B) Cell viability was determined after 4-PBA pretreatment (3 mM, 6 h). (C, D) 4-PBA inhibited up-regulation of Bip and CHOP in RAW264.7 cells induced by SiNPs. ^*^p < 0.05 vs Control, ^#^*p* < 0.05 vs SiNPs treatment group. Data are expressed as means ±SD of three independent experiments. **Table S1**. Real-time PCR Primer Pairs

## Data Availability

The datasets used and/or analyzed during the current study are available from the corresponding author on reasonable request.
