# Supplementary material for: Amorphous silica nanoparticles accelerated atherosclerotic lesion progression in ApoE−/− mice through endoplasmic reticulum stress-mediated CD36 up-regulation in macrophage
Source: Part Fibre Toxicol. 2020 Oct 2;17:50. doi: 10.1186/s12989-020-00380-0 (PMC7531166; doi:10.1186/s12989-020-00380-0)
Supplement: Supplementary file 1 — Additional file 1 Fig. S1. Histopathology alterations of lung tissues after intratracheal instillation exposure to SiNPs in ApoE−/− mice. n = 4 per group. Fig. S2. The body weight and food intake trends of mice. The body weight and food intake of the mice was measured weekly during the experiment. Data are expressed as means ± SD, n = 9. Fig. S3. Method for analyzing plaque area in aortic root. Regional errors in lesion size were avoided by acquiring sequential throughout of the entire aortic root. The lesion with the largest area was used for statistical analysis. Fig. S4 4-PBA inhibited the ER stress induced by SiNPs in RAW267.4 cells. (A) Cell viability was determined by MTT assay after 24 h-treatment with SiNPs. (B) Cell viability was determined after 4-PBA pretreatment (3 mM, 6 h). (C, D) 4-PBA inhibited up-regulation of Bip and CHOP in RAW264.7 cells induced by SiNPs. *p < 0.05 vs Control, #p < 0.05 vs SiNPs treatment group. Data are expressed as means ±SD of three independent experiments. Table S1. Real-time PCR Primer Pairs [file 12989_2020_380_MOESM1_ESM.pdf]

Supplementary material

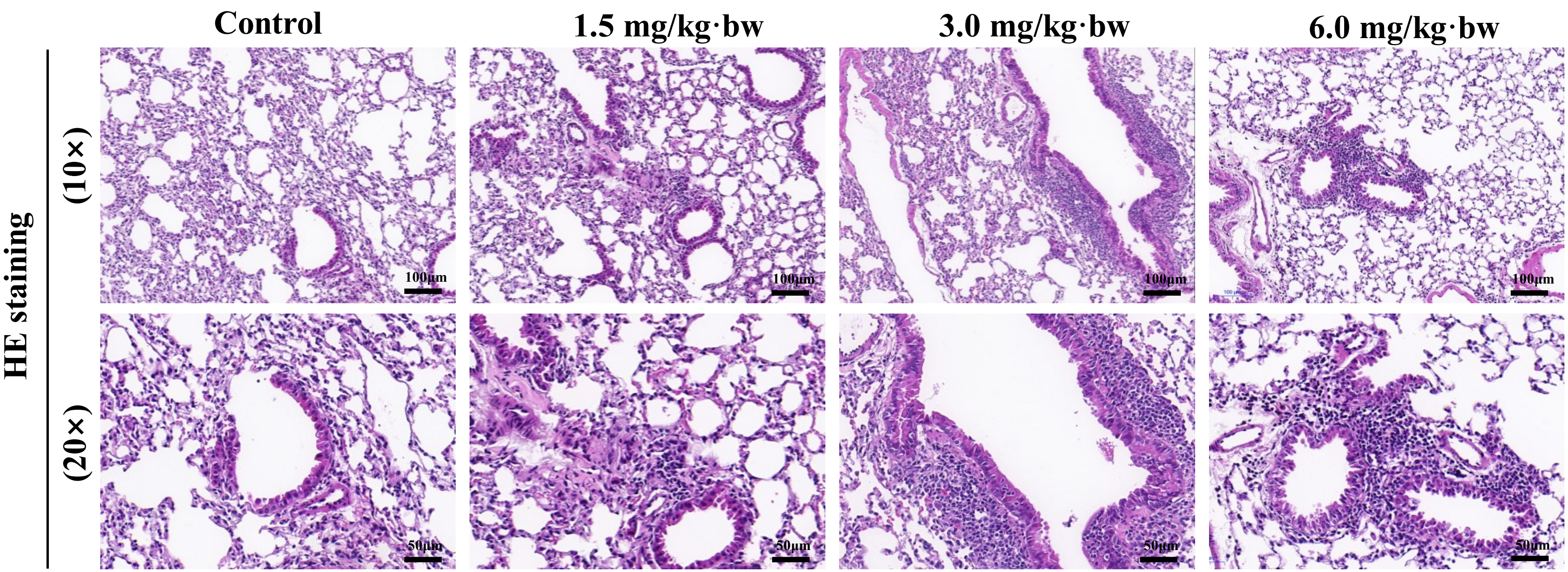

Fig. S1. Histopathology alterations of lung tissues after intratracheal instillation exposure to SiNPs in ApoE$^{-/-}$ mice. n = 4 per group.

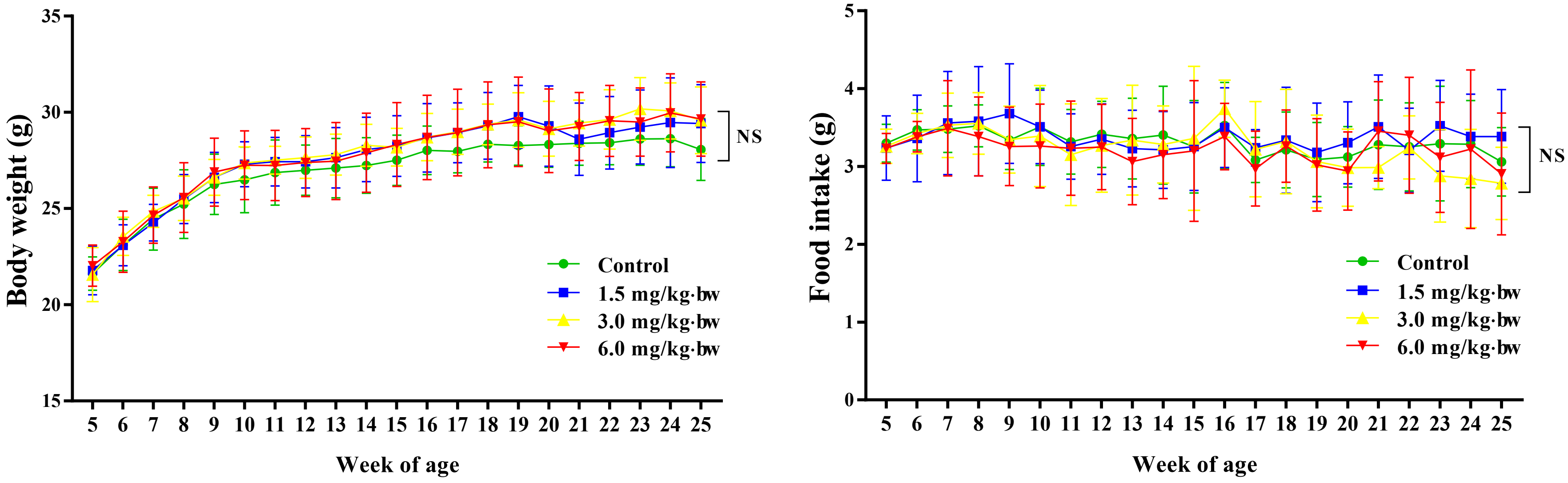

Fig. S2 The body weight and food intake trends of mice .The body weight and food intake of the mice was measured weekly during the experiment. Data are expressed as means ± SD, n = 9.

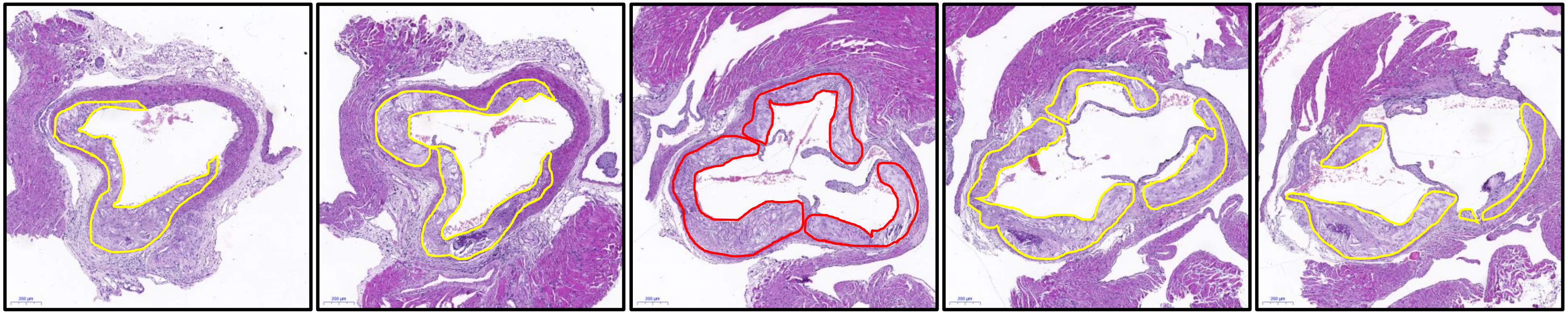

Fig. S3 Method for analyzing plaque area in aortic root. Regional errors in lesion size were avoided by acquiring sequential sections throughout of the entire aortic root. The lesion with the largest area was used for statistical analysis.

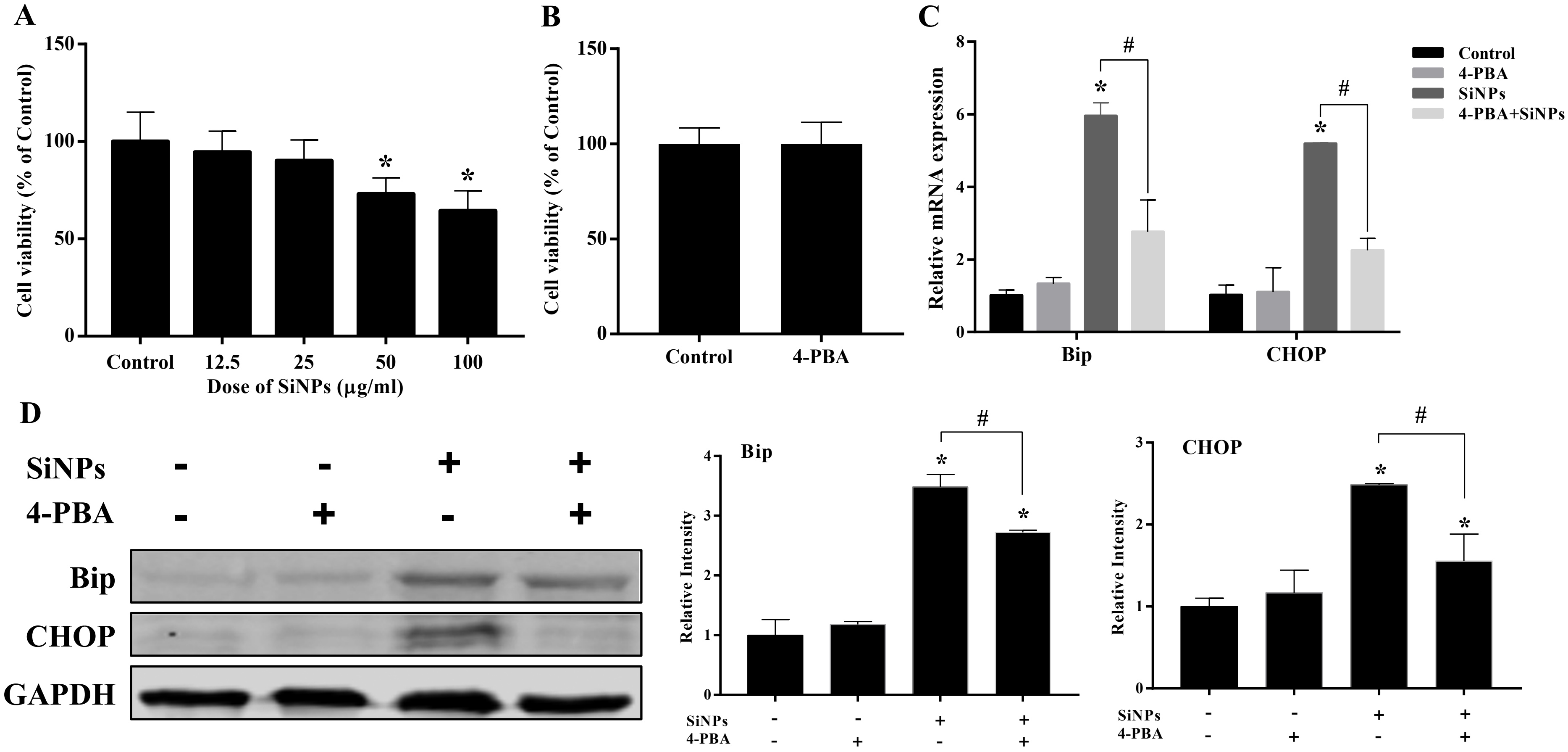

Fig. S4 4-PBA inhibited the ER stress induced by SiNPs in RAW267.4 cells. (A) Cell viability was determined by MTT assay after 24 hours-treatment with SiNPs. (B) Cell viability was determined after 4-PBA pretreatment (3 mM, 6 hours). (C, D) 4-PBA inhibited up-regulation of Bip and CHOP in RAW264.7 cells induced by SiNPs. **p*<0.05 vs Control, #*p*<0.05 vs SiNPs treatment group. Data are expressed as means ± SD of three independent experiments.

Table S1. Real-time PCR Primer Pairs

Gene.	Description	Primer sequence
**GRP78/Bip**	F	GTGTGTGAGACCAGAACCGT
	R	ACAGTGAACTTCATCATGCCG
**CHOP**	F	CCAGGAAACGAAGAGGAAGA
	R	CTTTGGGATGTGCGTGTG
**CD36**	F	GTGCTCTCCCTTGATTCTGC
	R	CTCCAAACACAGCCAGGAC
**SRA**	F	AGTCAGTGGATGGTGGGAGT
	R	GGAAGCCTGGTATTGTCTGG
**SRB1**	F	GCCTGTTTGTTGGGATGAA
	R	AGTCCGTTCCATTTGTCCAC
**ABCA1**	F	GGAAGGCACTCAAGCCACT
	R	CAGCCAACTCCTGAAAGGTC
**ABCG1**	F	TACCTGGATTTCATCGTCCTG
	R	AATGTCTGCTTTGCCTCGTT
**ACAT1**	F	AATGGACTCGGGCTGATTG
	R	AACCTCCGTCACTGTCTGCT
**β-actin**	F	GAGACCTTCAACACCCCAGC
	R	ATGTCACGCACGATTTCCC

Abbreviation: PCR, polymerase chain reaction.
